# Mapping special areas of the Brazilian National Malaria Control Program in the Amazon region: a territorial-based approach to surveillance

**DOI:** 10.1590/0074-02760240068

**Published:** 2025-03-31

**Authors:** Hermano Gomes Albuquerque, Gerusa Belo Gibson Santos, Alexandre San Pedro Siqueira, Ronan Rocha Coelho, Jefferson Pereira Caldas Dos Santos, Heitor Levi Ferreira Praça, Paulo Cesar Peiter, Leandro Henrique Vouga Pereira, Joseli Oliveira-Ferreira, Martha Cecilia Suárez Mutis

**Affiliations:** 1 Fundação Oswaldo Cruz-Fiocruz, Instituto Oswaldo Cruz, Laboratório de Doenças Parasitárias, Rio de Janeiro, RJ, Brasil; 2 Universidade Federal do Rio de Janeiro, Instituto de Estudos de Saúde Coletiva, Rio de Janeiro, RJ, Brasil; 3 Ministério da Saúde, Secretaria de Vigilância em Saúde e Ambiente, Coordenação de Eliminação da Malária, Rio de Janeiro, RJ, Brasil; 4 Universidade Veiga de Almeida, Laboratório de Inteligência Geográfica em Ambiente e Saúde, Rio de Janeiro, RJ, Brasil; 5 Fundação Oswaldo Cruz-Fiocruz, Instituto Oswaldo Cruz, Laboratório de Imunoparasitologia, Rio de Janeiro, RJ, Brasil

**Keywords:** malaria, surveillance, stratification, socioenvironmental determinants, Brazilian Amazon

## Abstract

**BACKGROUND:**

The malaria control strategy of the Brazilian Ministry of Health involves the classification of transmission contexts into special areas based on the distinct determinants of malaria in each location.

**OBJECTIVE:**

To search, find, organise, and map data about special areas using Brazilian databases and show their distribution among the states of the Brazilian Amazon.

**METHODS:**

A search related to the socioenvironmental determinants of malaria was conducted in Brazilian databases using the special areas of the Ministry of Health as a reference. Data were compiled by states in the Brazilian Amazon.

**FINDINGS:**

Indigenous areas occupy a significant portion of the Amazon territory and exhibit high incidence rates of malaria. Rural settlements also cover large areas of the Amazon, and in some states, more than 10% of malaria cases are associated with this typology. Legal and illegal mining areas, despite occupying small portions of the Amazon territory, contribute to the malaria caseload. In contrast, urban areas cover smaller regions, with low incidence rates.

**MAIN CONCLUSIONS:**

Despite the progress represented by the typological structure of special areas by the Ministry of Health’s, our findings reveal limitations related to them because of their complexities and emphasise the need to further substratify these areas to devise control strategies more adapted to them.

Malaria is a major public health challenge in many countries worldwide. In 2022, the estimated global malaria cases worldwide reached 249 million across 85 endemic countries.^1^ The Americas accounted for approximately 552,000 cases, concentrated in Amazon Rainforest.^1^ Within the Americas, Venezuela, Brazil, and Colombia were responsible for nearly 73% of all malaria cases in the region.^1^

Since 2014, the World Health Organization (WHO) has been revising its guidelines to focus on achieving malaria elimination. The objective is to eliminate all cases and sustain this scenario.^2^ Several documents have outlined ambitious goals, such as the “Global Technical Strategy for Malaria 2016-2030”,^3^ which envision a malaria-free world through the following targets: reduce global malaria incidence and mortality rates by at least 90% by 2030, eliminate the disease in at least 35 new countries, and prevent its reintroduction in countries that were malaria-free in 2015.

Two key documents underpin this study. The first is the “Framework for Malaria Elimination”,^4^ which serves as the basis for developing elimination plans tailored to local contexts and provides guidance on the tools, activities, and strategies necessary to achieve transmission interruption and prevent resurgence. The second is the “Malaria surveillance, monitoring & evaluation: a reference manual”,^5^ which aligns with the previous document and serves as a reference for strengthening malaria surveillance systems, national malaria programs, health information systems, and WHO officials advising countries on malaria surveillance.

In countries such as Brazil, where there is a significant ecological and vector-related distribution, social factors, population immunity, and different levels of healthcare programs implemented, the distinct malaria transmission contexts demand specific intervention strategies for each scenario.^4^ In this regard, the National Malaria Control Program (NMCP) is expected to implement different intervention strategies appropriate to each setting, which requires a stratification of the Brazilian territory, considering its complexities. According to the WHO, this stratification process should begin by distinguishing between receptive and nonreceptive areas, considering the determinants of malaria in the country.

Malaria receptivity is defined as an ecosystem with competent vectors, a suitable climate, and a susceptible population.^6^ This indicates an area in which conditions exist for *Plasmodium* spp. to transmit the disease. Therefore, aspects related to environmental and social determinants that affect malaria transmission should be considered when constructing receptivity models.^4^

Because of the high heterogeneity of human and environmental occupation in the Amazon, the NMCP has established typologies for disease transmission scenarios in Brazil, called “special areas”, for stratification. These include indigenous, mining activity, settlement project, rural, urban, and border areas.^7^ These areas exhibit differences in their transmission dynamics, requiring specific and targeted approaches for each context.

As a model to address territorial-based surveillance of malaria that overcomes the fragmentation of classical surveillance approaches (epidemiological, entomological, and environmental), we conducted a spatial descriptive study to characterise the Brazilian Amazon region by typologies of malaria transmission scenarios. This model also acknowledges that health and disease production processes are complex and socially determined.^8^

Therefore, this study aimed to map the special areas of the NMCP using indicators available in Brazilian databases and their distribution across the states comprising the Brazilian Amazon.

## MATERIALS AND METHODS

**Fig. 1:**
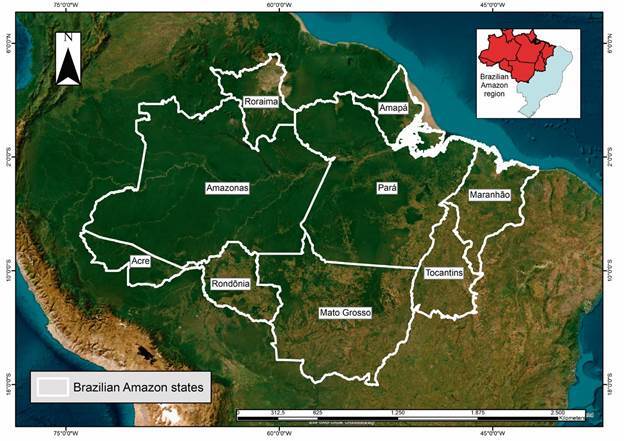
Brazilian Amazon and its states.

The Brazilian Amazon region covers nine states: Acre, Amapá, Amazonas, Pará, Rondônia, Roraima, Tocantins, Mato Grosso, and Maranhão (Fig. 1). This region covers an area of approximately 5 million square kilometres, representing approximately 58% of Brazil’s territory.^9^ Approximately 23 million people reside in this region, representing > 10% of the country’s population.

The special areas analysed in this study are the same as those used by the NMCP of the Ministry of Health^7^ (Table I). Note that the Ministry of Health does not use the secondary data analysed in this study to classify cases. To delineate specialised areas of malaria cases, Brazilian municipalities categorised these cases according to specific typologies, selecting from one of 44 categories available in the Malaria Epidemiological Surveillance Information System (Sivep-Malária). These selections were subsequently grouped into five designated specialised areas. The stratification of heterogeneity in the context of malaria transmission in the Amazon by the NMCP begins with the division between urban and rural areas. Then, the rural areas are divided into mining, settlement, indigenous, and other rural areas. We searched and organised the Brazilian databases for each of these typologies and mapped them in the Amazon region.

**TABLE I:** Details of the Brazilian databases related to special areas of the Brazilian National Malaria Control Program

Special areas	Description	Data source	Year of the last update
Indigenous territories	Consolidated data from indigenous territories as part of the Amazonian Network of Socioenvironmental Georeferenced Information for the Amazon	Digitised by the Socioenvironmental Institute from official documents based on data provided by the Brazilian Institute of Geography and Statistics (IBGE), Ministry of the Environment (MMA), and Indigenous National Foundation (FUNAI)	2020
Rural settlements	These are data on public lands allocated for agrarian reform, which is a series of measures aimed at promoting a more equitable distribution of land through changes in its ownership and use to respect the principles of social justice and increase productivity	Organised and digitised by the National Institute for Colonisation and Agrarian Reform (INCRA)	2021
Legal mining sites	The boundaries of the legal mining areas in the Amazon and their characteristics, such as the phase of activity, mineral substance, and company responsible	Organised and digitised by the National Mining Agency (ANM) - National Department of Mineral Production (DNPM)	2020
Illegal mining sites	The boundaries of illegal mining areas in the Amazon and their characteristics, such as data source, how the data were collected, and data collecting institution	Organised and digitised by the Socioenvironmental Institute	2020
Urban areas	Areas with > 10 buildings that were within the categories of interest of census tracts for urbanised areas, as well as those located < 3 km from these Census Tracts	Organised and digitised by the Brazilian Institute of Geography and Statistics (IBGE), Directorate of Geosciences, and Coordination of the Environment	2019
Rural areas	Indicator based on the sum of the “agricultural areas,” “anthropic areas in ecological tension,” and “dominant anthropic areas” classes, excluding the “urban influence areas” category from the latter class	Organised and digitised by the Brazilian Institute of Geography and Statistics (IBGE), Directorate of Geosciences, and Coordination of the Environment	2018

Indigenous areas are those permanently inhabited by indigenous people and those necessary for their way of life and social reproduction, as guaranteed by the 1988 federal constitution. The spatial database was digitised by the Socioenvironmental Institute using the Brazilian Institute of Geography and Statistics (IBGE) and the Ministry of the Environment database. The data have been updated until 2020. The number of indigenous areas and their size were calculated for each state of the Brazilian Amazon.

Another special area analysed was rural settlements, which are defined as a set of independent agricultural units, granted by the National Institute of Colonisation and Agrarian Reform (INCRA), where there was previously a private or public-owned rural property. The databases were extracted from the INCRA website. The data were updated until 2021. Similarly, for indigenous areas, the number of settlement areas and their extent were extracted for each state in the Brazilian Amazon.

Mining areas are defined by Decree No. 227, of February 28, 1967, in articles 6 and 709, and are those where different mining activities take place, which could be legal or illegal. The NCMP does not distinguish between legal and illegal mining areas in SIVEP-Malaria. Data on legal mining areas were extracted from the National Mining Agency, whereas data on illegal mining areas were compiled by the Socioenvironmental Institute. The data have been updated until 2020. The number of mining areas was extracted by type and state in the Brazilian Amazon. For legal mining areas, only those in the state of operation were considered in our analysis.

To determine the last class of rural areas, which involves farms and other classes of agricultural and livestock areas, the 2018 IBGE land use database was used. The indicator was calculated based on the sum of three classes: “agricultural areas”, “anthropic areas in ecological tension”, and “dominant anthropic areas”, except the “urban influence areas” category from the latter class. According to the metadata of this database, areas of ecological tension caused by human activity and anthropic areas dominated by human influence (excluding urban influence areas) comprise agricultural and livestock regions located in ecotones bordering urban and rural areas. According to the NMCP, rural areas, except mining, settlement, and indigenous areas, are mainly those used for agriculture or livestock, whether small or large scale.

Regarding urban areas, which are defined as areas with > 10 buildings located within census sectors, as well as those located < 3 km from these census sectors,^10^ the IBGE database from the “Brazil: Geographical Networks” collection, specifically the “Urbanised Areas” collection of 2019, was used.

To aggregate all indicators by state in the Brazilian Amazon, QGIS 3.28 software was used. The database used in this study is available at https://doi.org/10.35078/M2B248.

*Ethics* - The study used only open and available Brazilian databases.

## RESULTS

**Fig. 2:**
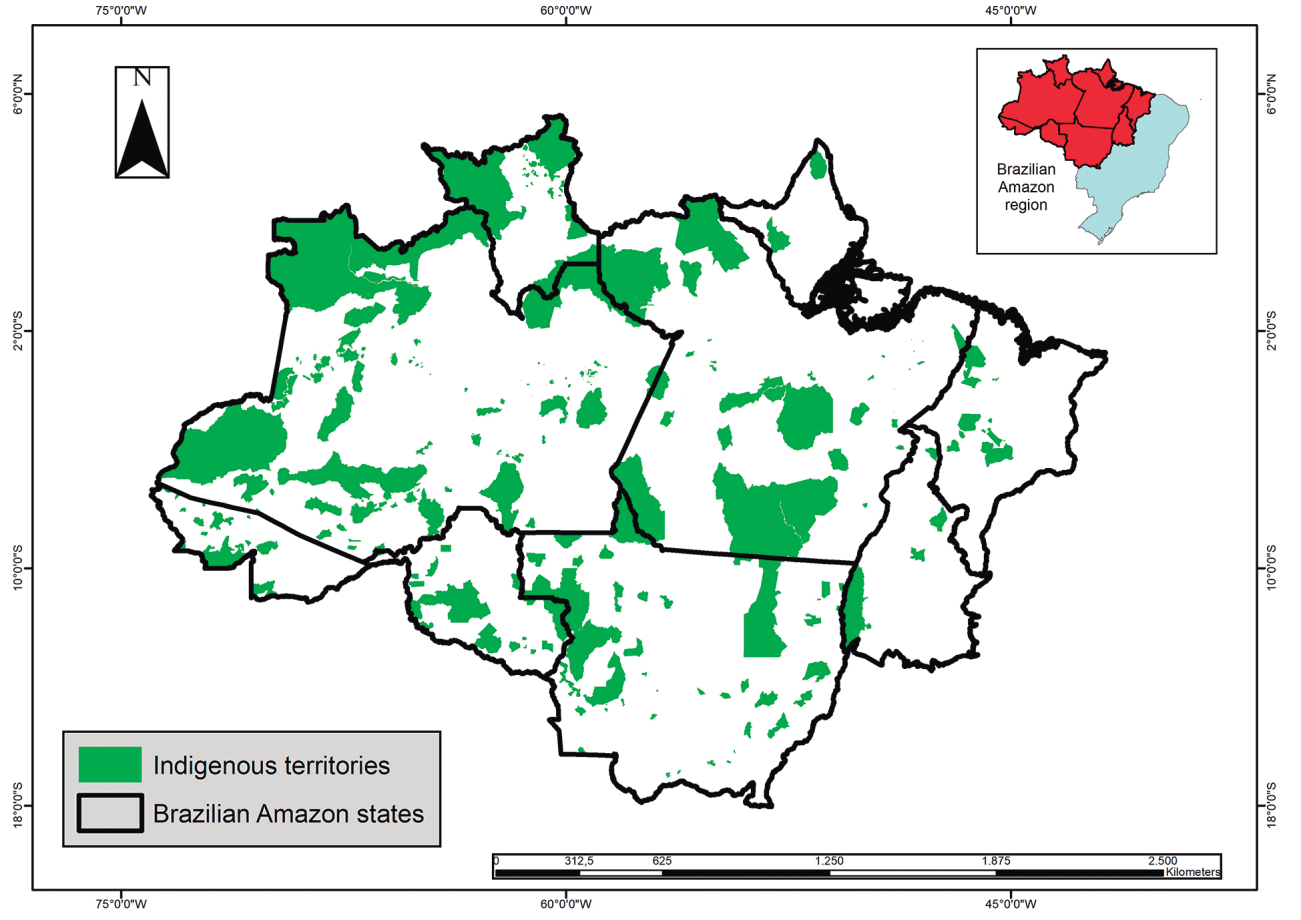
indigenous territories in Brazilian Amazon region.

**Fig. 3:**
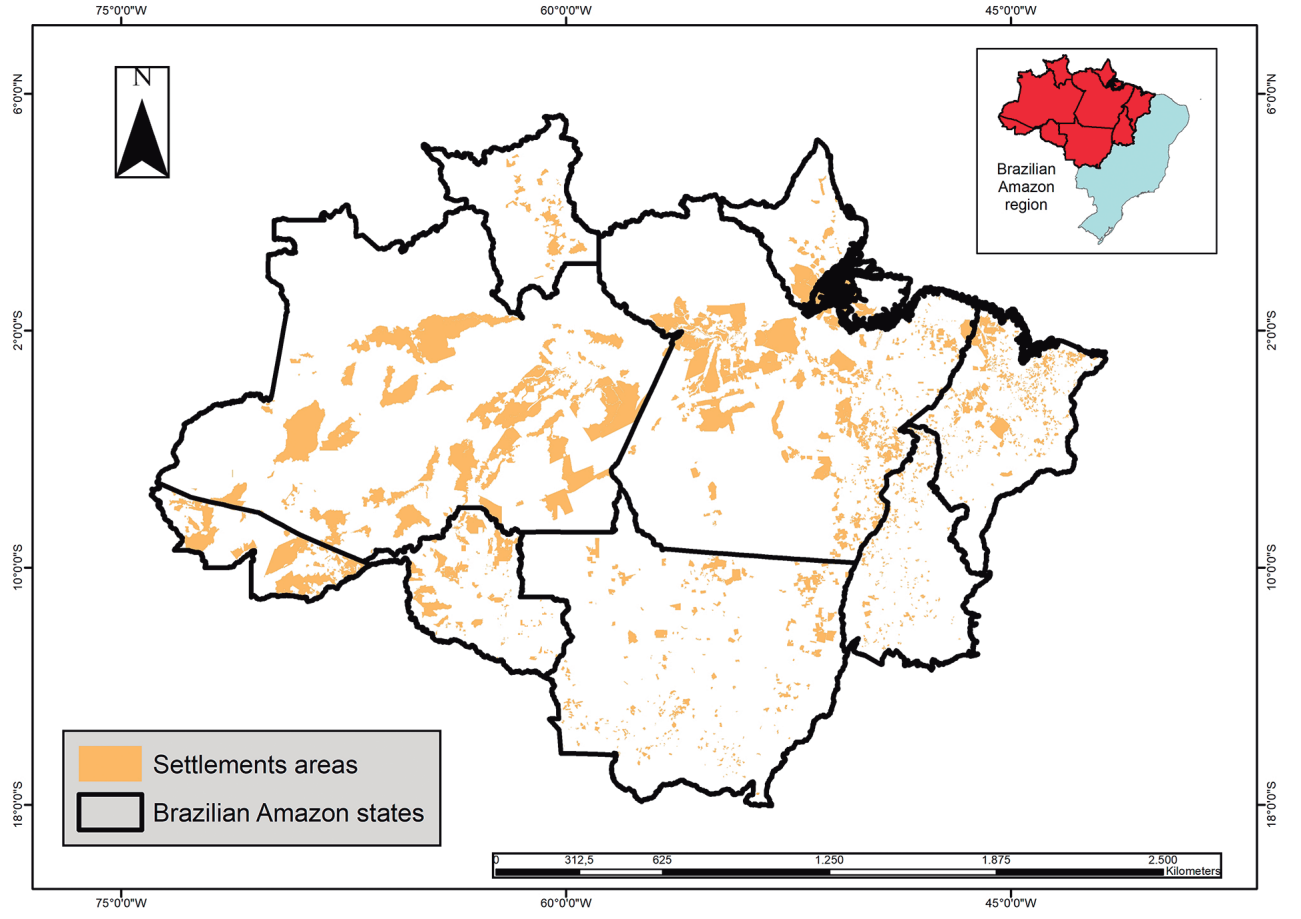
settlements sites in Brazilian Amazon region

**Fig. 4:**
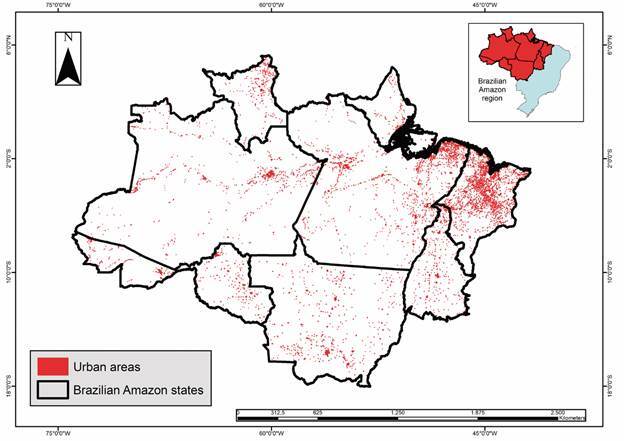
urban areas in Brazilian Amazon region.

**Fig. 5:**
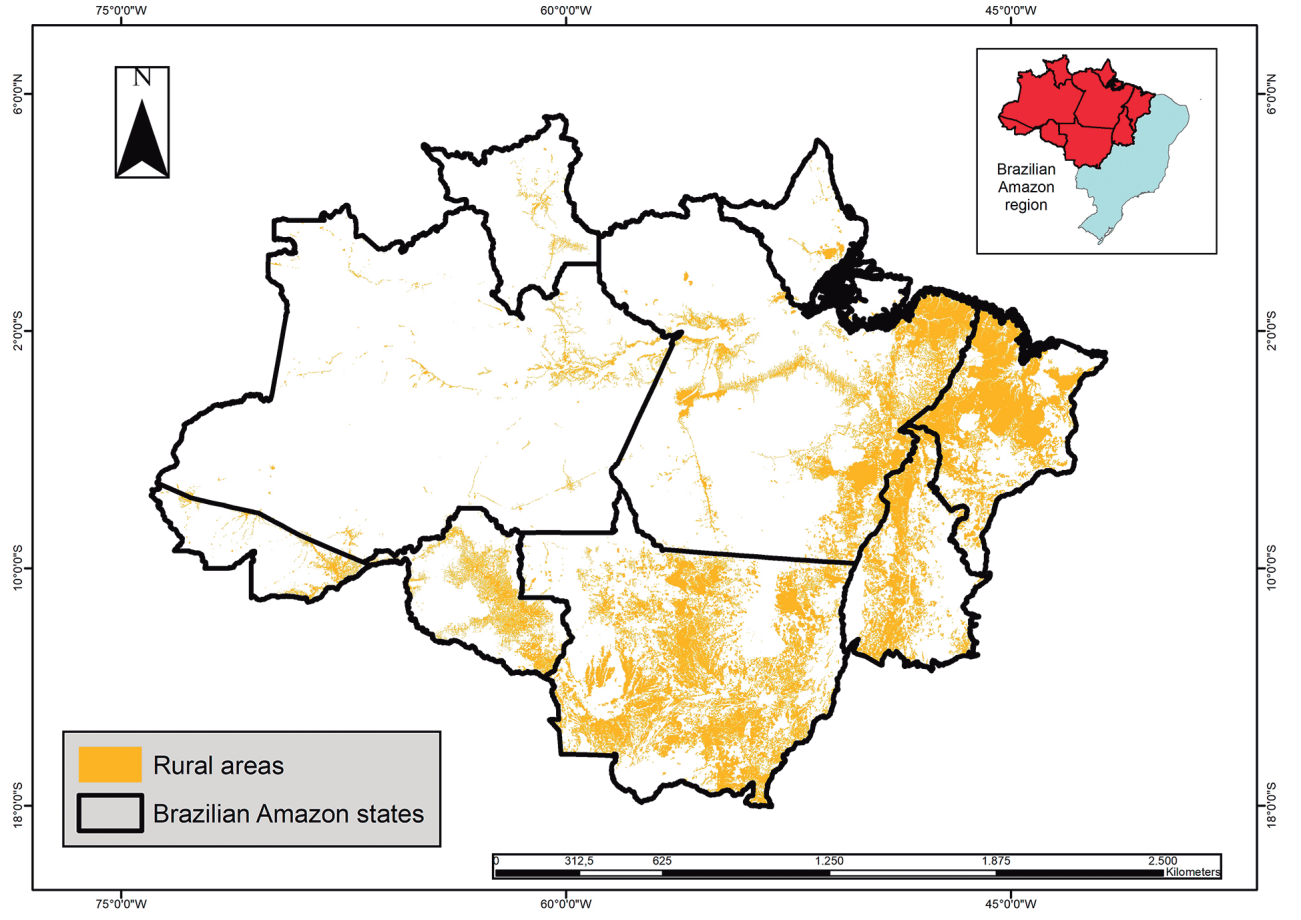
rural areas in Brazilian Amazon region

**Fig. 6:**
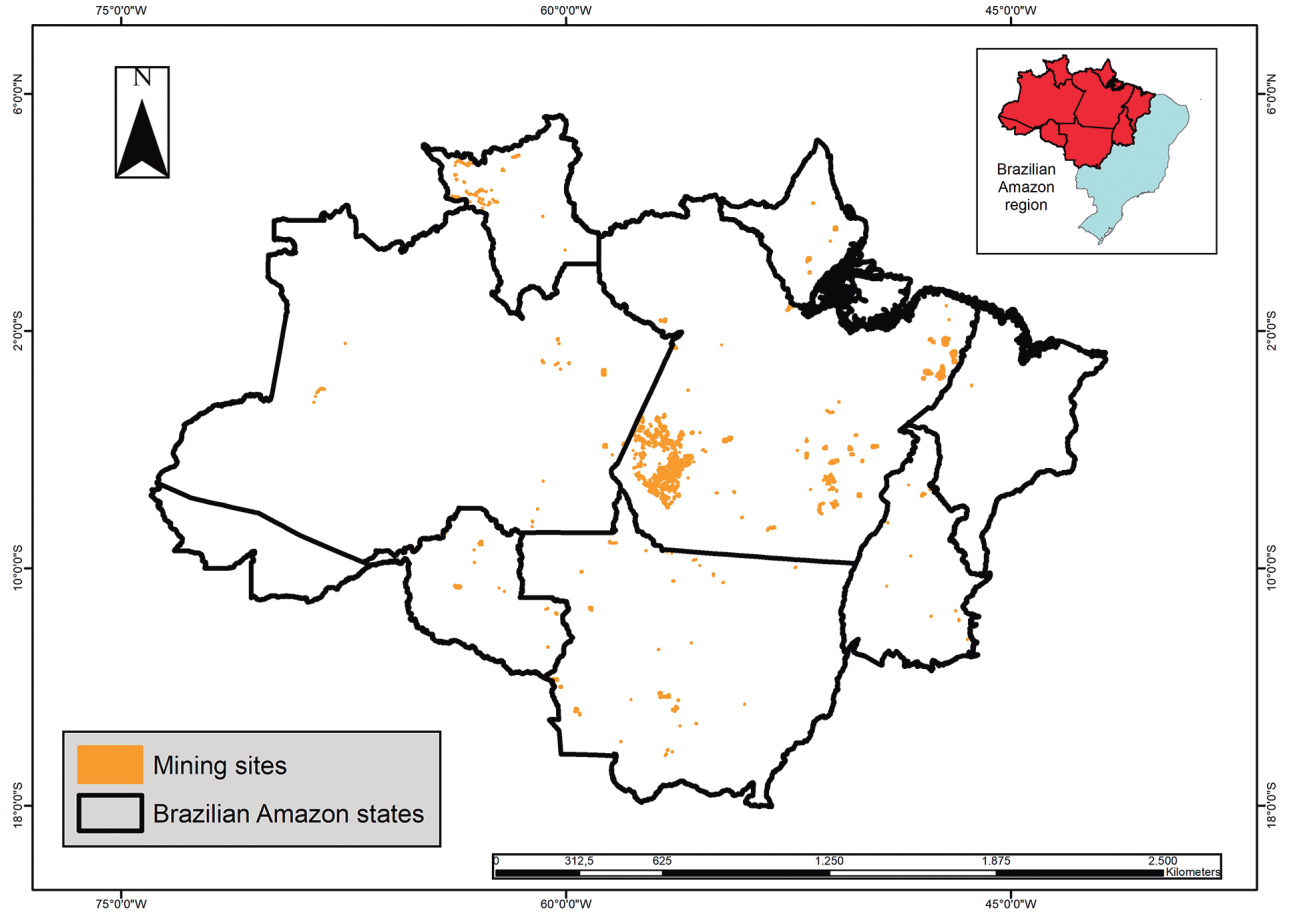
mining sites in Brazilian Amazon region

The Brazilian Amazon encompasses an area of 5,162,621.90 km² (with possible variations because of differences in the databases used). Indigenous areas (Fig. 2) occupy approximately 31.8% of this territory, accounting for 1,643,299.4 km², whereas rural settlements (Fig. 3) cover approximately 13.7% (709,894.60 km²). Urban areas (Fig. 4) accounted for 0.15% of this region (7,550.2 km^2^), whereas rural areas (Fig. 5) accounted for 17.2% (891,866.1 km²). Mining areas (Fig. 6), whether legal or illegal, represent a small percentage of the overall land, comprising 6,405.1 km² for illegal mining sites and 3,947.90 km² for legal mining areas (Table II).

**TABLE II:** Brazilian Amazon states and their corresponding indicators in relation to special areas of the National Malaria Control Program

Amazon states	Indigenous territories (Nº)	Indigenous territories (km²)	Rural settlements (Nº)	Rural settlements (Km²)	Illegal mining sites (km²)	Legal mining sites (km²)	Urban areas (km²)	Rural areas (km²)	Total states territories (km²)
Acre	52	25.045,9	130	60.828,3	0,0	0,0	222,2	13.862,2	156.625,9
Amapá	7	41.868,8	41	16.987,0	0,0	214,4	153,4	4.671,8	140.704,5
Amazonas	174	617.250,3	160	273.524,6	9,3	331,5	719,3	27.778,0	1.583.145,7
Maranhão	20	25.822,2	881	43.953,5	0,1	0,0	1.725,1	171.962,0	331.617,4
Mato Grosso	77	264.642,1	417	44.738,1	6,4	500,2	1.462,1	316.945,7	952.510,8
Pará	58	365.992,3	1058	204.460,3	6.218,3	2.643,7	1.854,6	202.105,4	1.240.524,0
Rondônia	29	80.599,3	223	40.947,5	152,2	125,4	570,4	56.123,9	246.473,1
Roraima	52	194.260,8	52	12.810,0	18,9	28,4	238,2	4.747,1	224.564,3
Tocantins	13	27.817,7	360	11.645,3	0,0	104,2	604,5	93.670,2	286.456,1
Total	482	1.643.299,4	3.322	709.894,6	6.405,1	3.947,9	7.550,2	891.866,1	5.162.621,9

^*^ Nº: number / Km² = square kilometres.

The results of the analysis of the indigenous areas by the states of the Brazilian Amazon (Fig. 2) revealed that the Amazonas has the largest number of indigenous land (n-174), and the largest extension of its territory was occupied by indigenous areas (617,250.3 km²) (Table II). This is followed by the State of Mato Grosso with 77 indigenous areas (264,642.18 km²) and the State of Pará with 58 (365,992.3 km²).

Rural settlements are predominantly concentrated in the states of Pará (Fig. 2), with 1,058 settlements covering an area of 204,460.3 km² of its territory, and Amazonas with 160 settlements covering an area of 273,524.6 km². The State of Maranhão has a significant number of rural settlements, with 881 settlements occupying 43,953.5 km² of its territory, and the State of Tocantins has 360 settlements, which cover an area of 11,645.3 km² (Table II).

Regarding mining areas, the State of Pará stands out in both legal and illegal mining activities, covering total areas of 6,218.3 and 2,643.7 km^2^, respectively (Fig. 2). Other states with notable mining activities include Rondônia, which has illegal and legal mining areas of 125.4 km², Mato Grosso with 500.2 km² of legal mining areas, and Amazonas with 331.5 km² of legal mining areas. The states of Acre and Amapá lack records of illegal mining areas, whereas Acre and Maranhão lack records of legal mining areas.

Regarding urban areas, Pará is the largest urban area among the states of the Brazilian Amazon, covering 1,854.6 km². It is followed by Maranhão, which has 1,725.2 km² of urban areas, and Mato Grosso, which has 1,462.2 km² (Fig. 2). Regarding other rural areas (farms and other classes of agricultural and livestock areas), the State of Mato Grosso is notably the largest, with an area of 316,945.7 km², followed by Pará, with 202,105.4 km², and Maranhão, with a total of 171,962 km² (Fig. 2).

## DISCUSSION

Malaria remains a significant cause of illness and death in endemic areas and has a significant effect on the health of the affected populations. The disease affects the most disadvantaged and vulnerable populations, emphasising the need for targeted control interventions that consider local contexts. The Ministry of Health’s approach to categorising malaria transmission into distinct typologies provides a fundamental framework for devising a range of control strategies tailored to suit the unique features of these regions.^7^ The main goals of this study were to search, find, organise, and spatially structure data related to special areas of malaria based on the guidelines used by the Ministry of Health. Furthermore, the development of spatial databases for special areas has the potential to address gaps in geographical information and mitigate spatial inaccuracies associated with the localities recorded in the SIVEP-Malária system. In the results of the initial examination, the Amazon region hosts the highest number of indigenous areas in the country. In 2021, they accounted for 46,111 (33.6%) malaria cases in Brazilian Amazon region,^11^ making them the second most affected special area, followed only by rural areas. According to the SIVEP-Malária,^11^ the State of Amazonas had the highest number of malaria cases in indigenous areas in 2021, with 23,105 malaria cases (and these areas covered 38,9% of its land).

Malaria is a significant cause of hospitalisations and deaths among some indigenous communities, particularly in children.^12^ Considering the extensive socioeconomic, geographic, and cultural diversity among these communities, the determinants of malaria in indigenous areas present notable diversity even among indigenous groups of the same ethnicity.^13^ Considering this epidemiological reality, managing malaria in indigenous communities requires recognition of their sociocultural diversity, greater vulnerability of this population, obstacles to accessing healthcare services in remote areas, availability of healthcare infrastructures, and implementing a structured disease control program tailored to the transmission dynamics of these ecosystems. This is important with regard to the epidemiological link between locations, factors, and groups involved in the transmission cycle of the disease. Therefore, a crucial step in devising effective strategies for these areas is to stratify them into subcategories and understand their determinants.

Rural settlement areas are associated with the Brazilian agrarian reform project and account for 7,703 (5.6%) malaria cases in the Amazon region (2021).^11^ Some states, such as Rondônia (1,782 malaria cases) and Acre (820 malaria cases), have > 10% of cases associated with this typology.^11^ These areas exhibit distinct transmission dynamics, with high infection rates in the early stages of land occupation, followed by a decline in rates over the years.^14^

Furthermore, rural settlements do not have uniform malaria transmission risks across their entire territories. Spatial analysis of malaria distribution in these settlements indicates the presence of specific risk factors frequently associated with land use,^15^ ^,^ ^16^ resulting in clusters of cases in certain areas. This heterogeneity in incidence may be correlated with the ecology of the malaria vector mosquito *Anopheles* spp. For example, the presence of *Anopheles darlingi* can vary because of forest cover percentage and forest edge density in settled landscapes.^16^ Deforestation also increases the average ambient temperature and could influence the development time from *A. darlingi* larva to adult, vectorial capacity of mosquito populations, and adult survivorship.^16^ ^,^ ^17^

Mining and gold mining areas in Brazil account for 20.542 (14.9%) malaria cases in the Amazon region^11^ and were the only special areas to experience an increase in both the number of cases and percentage of total cases from 2020 to 2021.^11^ Notably, the states of Pará (40.2%-8.027 cases), Amapá (30.2%-1.222 cases), and Roraima (25.0%-6.448 cases) reported a significant number of cases related to this typology. Although Amapá and Roraima do not have large mining areas according to the study databases, these areas exist. Therefore, the limitations of secondary databases must be understood, and the importance of their systematic updating highlighted. Furthermore, as Amapá and Roraima are in a border region (Roraima with Venezuela and Guyana, and Amapá with French Guiana), the population flow with neighbouring territories is high. The presence of mining areas not detected by information systems and the cross-border flows of miners from French Guiana and Venezuela result in the high incidence of malaria in Roraima and Amapá.^18^ ^,^ ^19^ Therefore, mining areas beyond Brazil’s boundaries must be analysed because this could attract populations to the area, subsequently increasing the number of malaria cases.

Malaria associated with mining activities, particularly gold mining, poses distinct obstacles that must be overcome to achieve successful malaria control in these areas. Because of illegal activities in mining areas, accessibility to healthcare services for diagnosis and treatment is often difficult,^18^ and this population is left without healthcare. The intense population movement in these areas, asymptomatic infections, and erratic self-medication also contribute to the emergence of drug resistance.^18^ ^,^ ^20^ ^,^ ^21^ In addition, mining areas cause water stagnation, which promotes the proliferation of the principal malaria vector *A. darlingi*.^20^

Urban areas accounted for 11.998 (8.7%) malaria cases in Brazilian Amazon region in 2021.^11^ In states with a higher number of urban areas, such as Mato Grosso, Pará, and Maranhão, the proportion of malaria cases was relatively low in 2021. These states are located in Meridional Amazon, also referred to as the arc of consolidated settlement, where large areas of soy farming, mining, and extensive livestock farming are prevalent.^22^ In these major urban centres where urbanisation is established, the Amazon biome is less prevalent, making it a less favourable environment for the main malaria vector *A. darlingi*.^23^

In 2021, the states of Rondônia, Acre, and Amazonas exhibited the highest number of urban malaria cases with 15.9% (2.219 cases), 14.6% (1.227 cases), and 11.1% (7.192 cases), respectively. Notably, these states are not among those with large urban areas (Table I). The Amazonas and Acre belong to the Western Amazon, an area that has been less affected by Brazil’s integration and development axes. This region has a well-preserved Amazon biome and extensive areas of national border.^22^ However, Rondônia has different characteristics, that is, it is located in the Meridional Amazon, where land use and occupation have been thoroughly consolidated, and the Amazon biome does not have the same level of conservation as in the other states. Thus, Brazil has different urban malaria scenarios with different transmission patterns.

Urban malaria in the Amazon region exhibits specific characteristics that warrant thorough analysis. Because of the heterogeneous nature of the urban environment, certain areas within cities are favourable for the presence of the malaria vector, facilitating the transmission cycle in those locations. Malaria typically occurs on the outskirts of urban centres and in areas with vegetation and agriculture within city limits.^24^ ^,^ ^25^

Urban expansion, resulting from population increase, migration, and displacement, along with territorial conditions favourable for vector proliferation, contribute to the establishment of urban malaria transmission cycles.^25^ The lack of proper planning in these peripheral urban areas, where housing is inadequate and basic infrastructure is insufficient, also contributes to the prevalence of malaria in these territories.^24^ ^,^ ^26^ Furthermore, most Amazonian cities are small urban spaces; thus, they have more characteristics of a rural environment than an urban environment.

In Brazil, Malaria commonly emerges in rural settings where the main malaria vector is found.^16^ ^,^ ^17^ ^,^ ^23^ In 2021, rural areas accounted for 37.0% of all malaria cases in Brazil, comprising a total of 50,842 cases. Moreover, considering cases in indigenous areas, mining areas, and settlements frequently situated in rural regions, approximately 85% of Brazilian cases are linked to the rural typology.^11^ In terms of territory, roughly one-fifth of the Amazonian states are occupied by farms and other classes of agricultural and livestock areas, with approximately 890,000 km^2^ in the region.

Therefore, rural malaria is more prevalent in areas with inadequate housing, inadequate infrastructure, and an abundance of breeding sites that increase human-vector contact.^27^ Significant heterogeneity exists within rural malaria categories. An understanding of the macroregion^22^ where it is located may help in the subcategorisation of Brazilian rural areas. In addition, the time elapsed since the beginning of rural area structuring may indicate different levels of exposure to the disease transmission cycle because rural areas in the early stages of development often have a higher number of malaria cases than consolidated areas.^14^ ^,^ ^28^

In general, the classification of malaria into special areas by the NMCP of the Ministry of Health has emerged as a crucial strategy for understanding the epidemiological patterns of malaria in Brazil. Each special area has a unique context that requires different approaches, such as control measures, surveillance activities, and public policy development. However, these typologies underestimate the diversity of malaria transmission within a specific area. The implementation of new categories for special areas will provide novel perspectives on surveillance, control actions, and formulation of more specific public policies tailored to their respective contexts.

Regarding the distribution of special areas across states, Pará has the most extensive urbanised area and the largest mining area (both legal and illegal) in the Brazilian Amazon region. Mato Grosso has the largest rural area (farms and other classes of agricultural and livestock areas), whereas Amazonas has the largest indigenous and settlement areas. Pará has the highest number of settlements in the Amazon. Analysing these typologies in an integrated manner is crucial because it involves considering indigenous and mining areas together and acknowledging their collective contribution to the heightened malaria potential in the affected areas.

In special areas, indicators of the socioeconomic and environmental determinants of malaria can be a valuable tool in the development of intersectoral actions in the health sector. Different government sectors are responsible for managing each of these special areas, whether at the federal, state, or municipal levels.

In each state and municipality, the determinants influence malarigenous areas, defined as specific and circumscribed localities within a malaria-endemic area that contain continuous or intermittent epidemiological factors necessary for malaria transmission.^29^ This understanding will empower the health sector to ensure the allocation of resources and target at-risk areas, ultimately leading to better surveillance and control of malaria in the Brazilian Amazon region.

Finally, the relationship between malaria determinants and their dynamics and distribution must be elucidated, establishing the regionalisation of micro (local scale) and macro (regional scale) malaria foci in the Brazilian Amazon region according to their determinants. This will allow for the consideration of multiple malaria territories in the country. Subsequently, the development of new political regionalisation, with intermunicipal and interstate agreements, will be feasible, focusing on the macro and micro malaria regions of the Amazon and structuring a new regional governance approach for malaria elimination in Brazil.^30^
